# The epidemic forest reveals the spatial pattern of the spread of acute respiratory infections in Jakarta, Indonesia

**DOI:** 10.1038/s41598-024-58390-3

**Published:** 2024-04-01

**Authors:** Yuki Novia Nasution, Marli Yehezkiel Sitorus, Kamal Sukandar, Nuning Nuraini, Mochamad Apri, Ngabila Salama

**Affiliations:** 1 Department of Mathematics, Faculty of Mathematics and Natural Sciences, Institut Teknologi Bandung, Bandung, 40132 Indonesia; 2 Department of Mathematics, Imperial College London, London, SW7 2RH United Kingdom; 3 DKI Jakarta Provincial Health Office, Jakarta, Indonesia

**Keywords:** ARI, Epidemic forest, Early detection, Diseases, Health care, Mathematics and computing

## Abstract

Acute respiratory infection (ARI) is a communicable disease of the respiratory tract that implies impaired breathing. The infection can expand from one to the neighboring areas at a region-scale level through a human mobility network. Specific to this study, we leverage a record of ARI incidences in four periods of outbreaks for 42 regions in Jakarta to study its spatio-temporal spread using the concept of the epidemic forest. This framework generates a forest-like graph representing an explicit spread of disease that takes the onset time, spatio-temporal distance, and case prevalence into account. To support this framework, we use logistic curves to infer the onset time of the outbreak for each region. The result shows that regions with earlier onset dates tend to have a higher burden of cases, leading to the idea that the culprits of the disease spread are those with a high load of cases. To justify this, we generate the epidemic forest for the four periods of ARI outbreaks and identify the implied dominant trees (that with the most children cases). We find that the primary infected city of the dominant tree has a relatively higher burden of cases than other trees. In addition, we can investigate the timely ($R_t$) and spatial reproduction number ($R_c$) by directly evaluating them from the inferred graphs. We find that $R_t$ for dominant trees are significantly higher than non-dominant trees across all periods, with regions in western Jakarta tend to have higher values of $R_c$. Lastly, we provide simulated-implied graphs by suppressing 50% load of cases of the primary infected city in the dominant tree that results in a reduced $R_c$, suggesting a potential target of intervention to depress the overall ARI spread.

## Introduction

Acute Respiratory Infection (ARI) is an infection that affects the respiratory tract and is classified as upper and lower respiratory tract infections. The common symptoms may include fever, cough, wheezing, and short-rapid breathing^1^. ARI, including pneumonia and influenza, has become a global health burden, particularly for infants and elder people^2–6^. In 2019, ARI caused 4.43 % of deaths worldwide for all ages^7^, and at the end of the year, the infection caused by the coronavirus disease (COVID-19) became a global pandemic. The infections of ARI during the pandemic COVID-19 tend to decline in most regions due to the implementation of public health measures^8–10^. However, some studies show the increasing burden caused by respiratory viral infections in several countries after the pandemic^11–13^.

Various bacteria and viruses can be the cause of ARI. Physical contact and airborne routes are ways for the infection to be transmitted from the infected individual to the susceptible ones. The mobility of people is one factor that causes the spread of infection from one region to another^14^. The study of the spread of infection is an attempt to control the outbreak and relieve the burden on health institutions. Some studies model the spread of infection in both temporal and spatial aspects. For example, the spread of COVID-19 had been studied through temporal model^15,16^ which incorporated human mobility^17^, limited testing capacity^18^ and provide information for public policy making^19–21^. The spatio-temporal aspect in the spread of COVID-19 had also been modeled for cases in South America^22^, Bangladesh^23^, Iran^24^, Italy^25^, and Singapore^26^. The spread of respiratory infections between regions has been modeled by network concept through traffic connection^14^ and airline route^27–30^.

The concept of epidemic tree was introduced to understand the spatial pattern of disease spread, particularly the 2001 UK foot-and-mouth outbreak^31^. In the research, an algorithm was constructed to determine the source of infection of each case which arises in several pig farms within the duration of the outbreak. The epidemic forest approach was constructed to spasialize epidemic modeling which was able to identify primary cases at the individual-level and built the forest for the 2013 dengue fever epidemic in Guangzhou, China^32^.

The identification of primary cases is also important for region-scale epidemic modeling. It can provide information for early detection of an outbreak which may occur in a certain city or country. Therefore, a better understanding of the disease spread in region-scale is crucial to build awareness and preparation by the government to control the outbreak. In this study, we developed an epidemic forest model to describe the spread of infectious diseases between regions by adopting an inter-individual spread model. Through adoption, a region is assumed to only receive transmission of infection from one neighboring region, which in terms of spread between individuals represents close contact. Thus, a unique source of infection can be determined. This study aims to identify the origin of ARI’s spread in the Special Region of Jakarta from 2017-2021 by observing the consistency of seasonality and severity of the disease at the district level for early detection using epidemic forest.

## Results

The dataset comprises monthly records of ARI incidences from 2017 to 2021 for 42 regions in Jakarta. These records were collected from hospitals and health care centers in each corresponding region that we assumed represented the actual situation of ARI incidences.

### A data-based evidence of ARI’s seasonality

**Figure 1:**
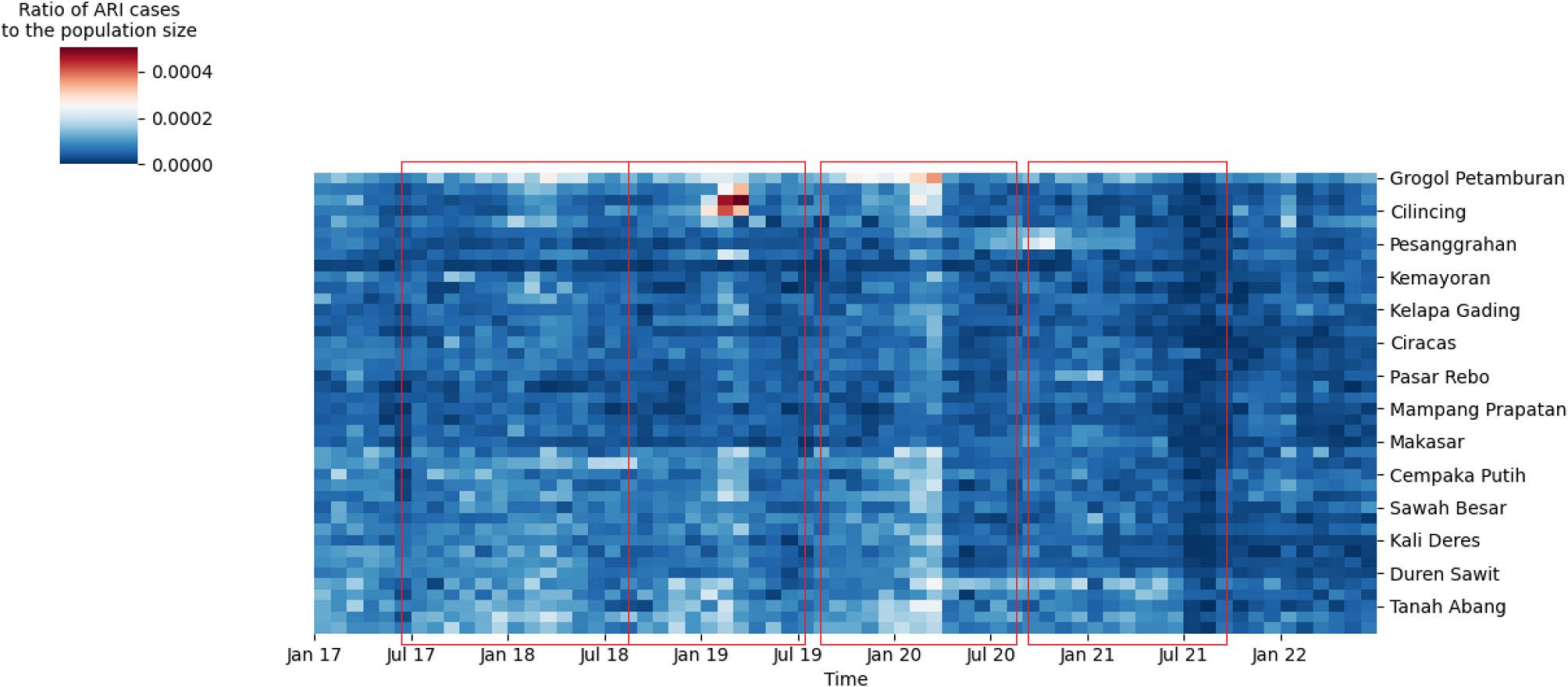
ARI outbreak occurs seasonally, identified from the heatmap data: The heatmap shows the ratio of ARI cases to the population size in 42 regions in Jakarta, with a median of 168 cases across regions. Due to scale limitation, the heatmap explicitly indexes only seven regions on the y-axis. We found four ARI outbreaks denoted by red boxes. For each period, all regions consistently experienced increased cases marked with white-to-red grids as they peaked, followed by decreased cases at the end of the outbreak period.

We investigated the spatio-temporal dataset of ARI incidences by the heatmap-style diagram, with the row representing the list of the observed regions. To cluster regions with similar ARI incidences, we used hierarchical clustering based on the ratio of ARI cases relative to the population size. Shown in Fig. 1, we identified four periods of outbreaks marked by the peaks of ARI incidences roughly in all 42 regions, with (1) period 1: July 2017 - July 2018 (2) period 2: Aug 2018 - July 2019, (3) period 3: September 2019 - Aug 2020 and (4) period 4: September 2020 - September 2021. For each period marked by red boxes, the ratios gradually increase from around zero (blue grids) to white-to-reddish colored grids as it reaches the peak of the outbreak, before it decreases back to zero. In other words, ARI incidences roughly follow Gaussian-like curve centered around the peak time of the outbreak. While we inferred the first period to last as long as 14 months, the other three periods were consistent in about 11-12 months, suggesting an annual ARI outbreak.

In addition, Fig. 1 shows that some regions constantly had more cases than others across periods, such as regions from Makasar Region downward, or a small cluster at the top of the diagram. Specific to the first three periods, these region cohorts experience an earlier increase of cases than the rest marked by whitish grids at the start of each period in Fig. 1. This result suggests that some regions that experienced the outbreak earlier potentially contributes to the infection of the neighboring regions. Though hierarchical clustering is able to identify the seasonality of ARI outbreaks, this method does not provide results on the explicit spread of the disease among regions.

### The inferred onset time negatively correlates the load of cases across regions

A region is classified as infected if the cumulative number of cases exceeds the threshold value. In this case, we set the threshold of the outbreak as 20 cases per 100,000 population In other words, regions are called infected by ARI outbreak if the total cases recorded per period exceeds 0.02% of the total population. Per infected region, we record its onset as the first time of the occurrences of the outbreak. This concept of onset resembles that for individual cases in clinical data – onset time is defined as the first time a person develops disease symptoms. This measure of the outbreak is essential to infer the spatial behavior of the spread of ARI, with those having earlier onset potentially infecting neighboring regions.

**Figure 2:**
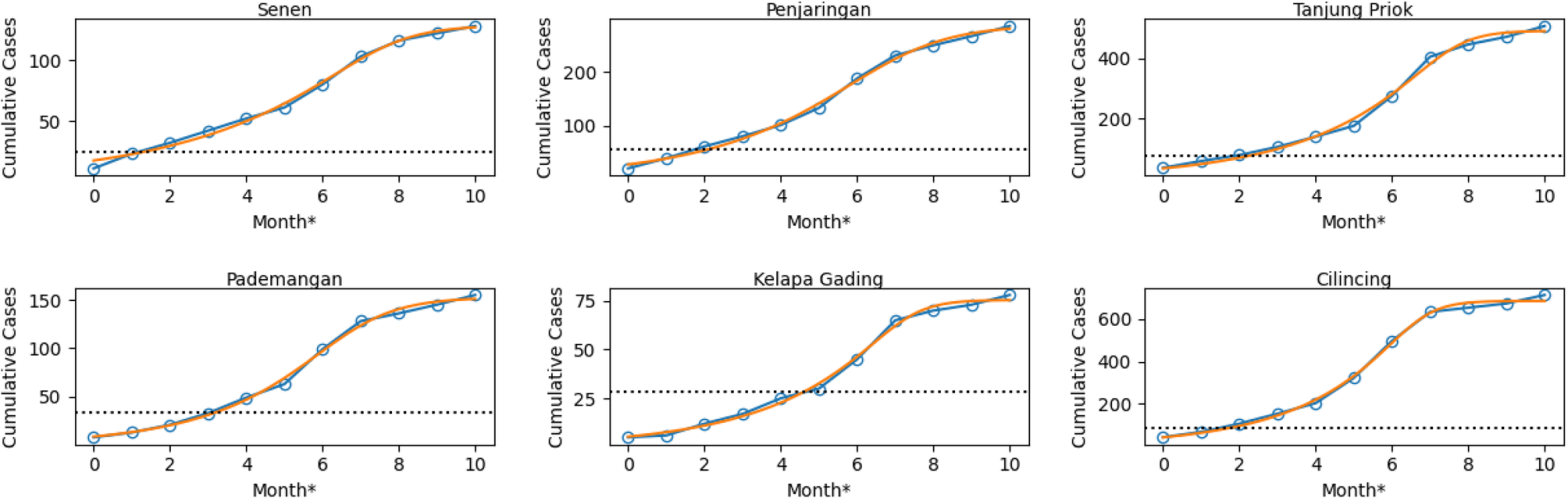
The inferred onset time of the outbreak: the logistic curves well-fit the cumulative data of ARI incidences across regions with the snippets of the comparison shown for six regions in Period 2. The inferred onset time of the outbreak is the intersection between the fitted curve (orange line) and the actual data (blue dots). The threshold varies among regions depending on the population size–regions with higher population size require more cases to reach the outbreak status.

In practice, we first calculated the cumulative version of the ARI incidences per period that generally resulted in S-curved trends across regions. We obtained the explicit formulas of cumulative case data using a generalized logistic curve or Richard’s curves. The onset time of the outbreak for each region was directly determined by identifying the intersection between the fitted curve and the threshold line. We rounded the estimated onset times to integers to match our study. Per period, the estimated onset time will be either 0 or positive integers, with an onset time 0 representing regions with the earliest outbreak onset. Figure 2 shows the snippet comparing the actual cumulative ARI incidences with the fitted logistic curves. For the six observed regions in Period 2, the logistic curves behave in the same manner as the actual data and allow us to estimate the onset time of the outbreak. Relative to the other five observed regions, Kelapa Gading and Pademangan Region took more time for the data to exceed the threshold, indicating a later outbreak onset than others. In contrast, though Cilincing Region has a higher threshold 85 total cases to reach the outbreak, the cumulative data rapidly increased and exceeded the threshold before the first two months from the 0 reference month. From only these six observed cases, we found a significant difference in the inferred outbreak onset, indicating some regions contribute to the case prevalence of others.

**Figure 3:**
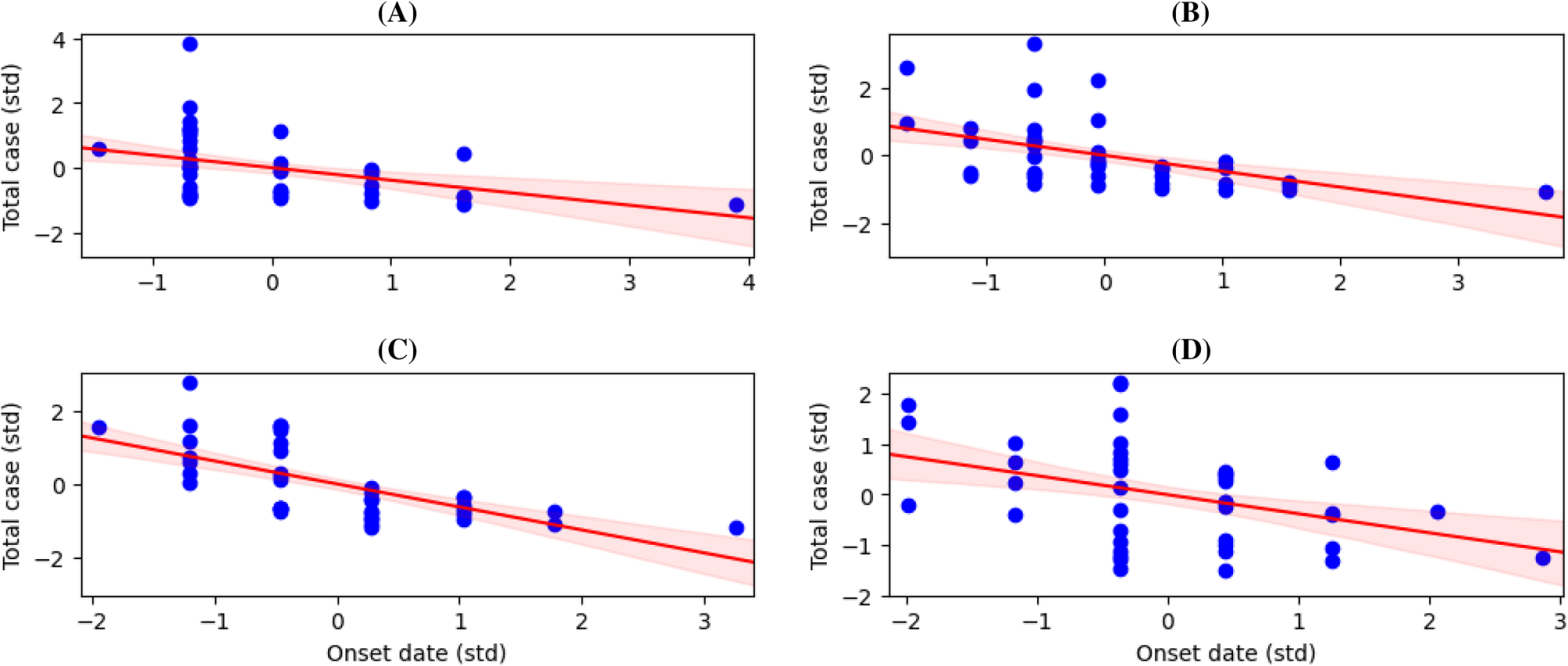
Onset times are negatively associated with the load of cases across all periods: we constructed a linear model that links the inferred onset times with the total cases for Period 1-4 (**A**)–(**C**). To ease the inference of slopes and intercepts of the models, we standardized the onset time and total cases data depicted by blue scatters. The model suggests a negative association between the onset time and load of cases, which is captured by the model mean and its 95%-credibility interval.

To enhance our study, we investigated the inferred onset time and found that it is negatively associated with the total load of cases. Figure 3 shows consistent decreases in total cases as the onset time increases across outbreak periods. Given the evidence that highly-infected regions start the outbreak earlier than others, one plausible scenario is that the overall infections start from the culprits and expand to other regions through human mobility. Since the culprits must be that with the earliest onset time, Fig. 3 suggests that culprits are those highly-infected regions. Though this analysis does not provide an exact spatial pattern of disease spread, it may indicate that culprits are the precise target of intervention to depress the overall spread of ARI disease in Jakarta. These inferred onset times were further leveraged in the next section to construct the forest-like graph of disease spread.

### The epidemic forest infers a robust spatial pattern of ARI spread across weight combination

By the concept of the epidemic forest, we leveraged the inferred onset times to construct the spatial pattern. The pattern is represented by tree-like graphs that depict how the disease spread within the set of regions. We defined that an infected region can infect others, with one that infects termed as a parent while those infected are termed as children. For each region, we look for the neighbouring areas and select one as the parent with the highest value of strength of linkage (SoL). Aside from the onset time, we proposed to consider at least three additional aspects to determine the parent of children regions: (1) spatial distance, (2) temporal distance, and (3) case prevalence, which are weighted by $W_s, W_t$ and $W_p$, respectively. More about the detail of the construction of the epidemic forest is described in the Methods section. In this study, we tested eight combinations of the weights to assess how sensitive the implied graph is due to the changes in the combination of weights. Table 1 lists all combinations of the weights that we are interested in assessing.

**Table 1:** The eight selected combinations of $W_s, W_t$ and $W_t$ weigh the spatial, temporal distance, and case prevalence to calculate the strength of linkage.

Combination of weights	Spatial distance weight ($W_s$)	Temporal distance weight ($W_t$)	Case prevalence weight ($W_p$)
1	1/3	1/3	1/3
2	0.10	0.45	0.45
3	0.45	0.10	0.45
4	0.25	0.50	0.25
5	0.15	0.70	0.15
6	0.45	0.45	0.10
7	0.25	0.25	0.50
8	0.15	0.15	0.70

We assume that these weights sum up to one, with higher weights representing the higher effects of the particular aspect.

**Figure 4:**
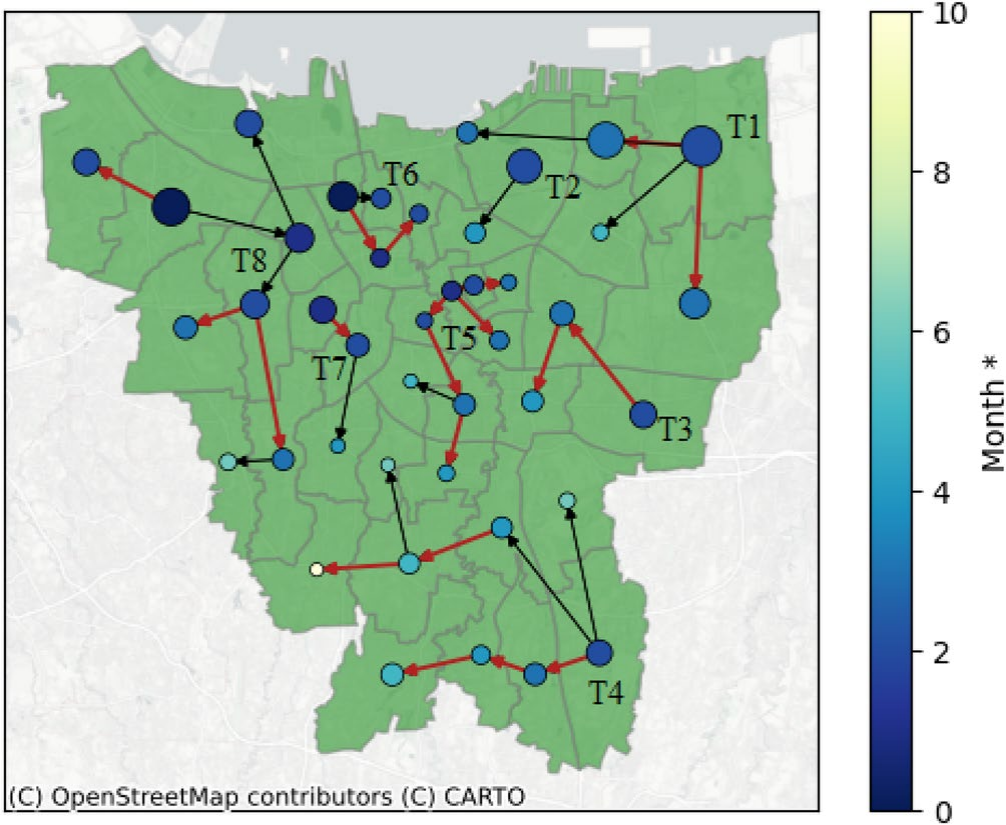
Resulting epidemic forest for Period 2 with combination 2. The red edges are set of edges which always appear in all combinations with $W_s < 1/3$ (All Data sets and Python code are available on GitHub (https://github.com/marliyehez/Epidemic-Forest) with the source of the map from^33^).

Special Region of Jakarta is a metropolitan region with a well-developed road network and transportation system so $W_s$ for spatial aspects is selected to be non-dominant compared to two other weight values. In this regard, we choose the combinations that comply $W_s < 1/3$. Figure 4 shows the epidemic forest for period 2 with combination 2. The size of each node at Fig. 4 indicates the case prevalence of the node, the bigger nodes mean a bigger number of case prevalence. The color of the node represents the onset time of the outbreak, i.e., the darker shade of blue indicates the earlier onset time and vice versa. From Fig. 4 we obtain eight trees, i.e *T*1, *T*2, ..., *T*8. The tree which is notated by *T*5 has the most children cases among others, which is called as dominant tree. We observe that the primary case at the dominant tree has a smaller size of node compared to the other tree, which shows that the spread size is not only affected by prevalence. The red edges in Fig. 4 indicate a set of edges that also appear in all combinations with $W_s < 1/3$. The intersection set of edges in Fig. 4 will act as a reference in this process and is called $E^*$.

**Figure 5:**
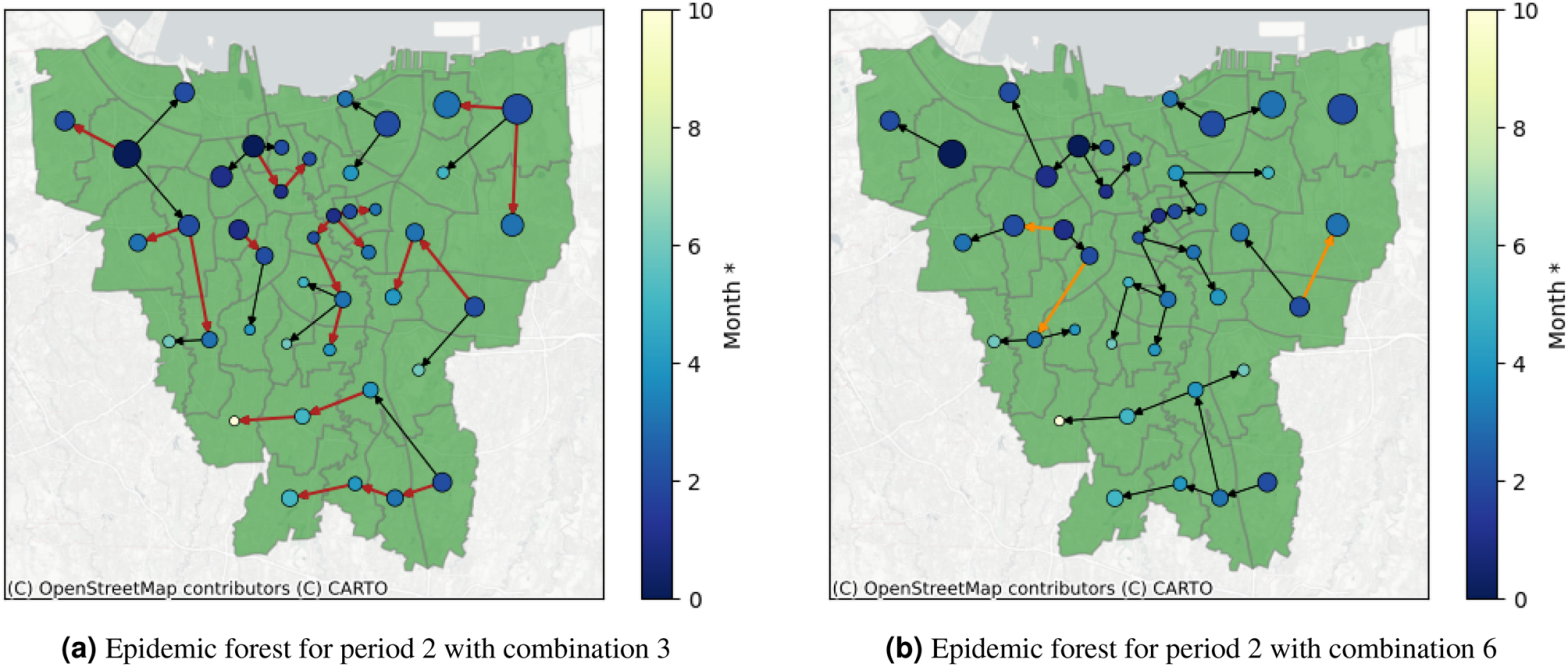
Comparison between resulting epidemic forest between non-dominant temporal aspect in (**a**) and non-dominant prevalence aspect in (**b**).

Next we will perform sensitivity analysis for temporal and prevalence aspects and we use period 2 for illustration. For temporal aspects, if the weight $W_t$ is replaced to be non-dominant, for example in combination 3, $E^*$ will always appear in the epidemic forest as shown by red edges in Fig. 5a. This result indicates that temporal aspects are not sensitive since their changes do not affect $E^*$. As for prevalence aspects, if $W_p$ is changed to be non-dominant, for example in combination 6, $E^*$ does not always appear in the resulting epidemic forest. This indicates there is sensitivity against the changes in $W_p$. The comparison of sensitivity analysis for both aspects is shown in Fig. 5.

**Figure 6:**
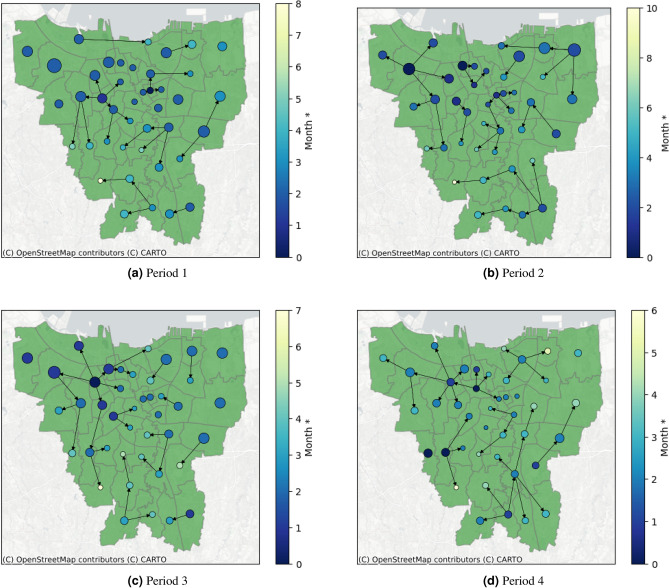
Result of epidemic forest for all period.

Based on sensitivity analysis, relatively low $W_p$ can eliminates few edges that ought to be uphold since it appears in $E^*$ as seen in 5b we obtain orange edges which do not appear in $E^*$. The selected $W_t$ in combination 7 also does not affect the edges on the resulting epidemic forest in $E^*$. Hence, we have two combinations with relatively large $W_p$, that is combinations 7 and 8. We chose combination 7 for the weights in Period 2 considering the discrepancies between weights are not large. The search for the best weight combination for all periods is presented in Table 2 and the resulting epidemic forest for each period is presented in Fig. 6.

**Table 2:** Selected weight combination of all three factors for *SoL* calculation at each period.

Period	$W_s$	$W_t$	$W_p$
1	0.15	0.15	0.70
2	0.25	0.25	0.50
3	0.25	0.25	0.50
4	0.25	0.25	0.50

Figure 6 shows that at each period, the dominant tree in the forest has at least eight from total 42 districts. Despite being the most dominant one, these trees do not always have a significantly greater number of child cases compared to other trees. For example, in Period 2 as shown in Fig. 6b, the dominant tree has eight child cases even though two other trees have seven child cases per tree. We observe that in periods 1 and 3, the primary cases of the dominant tree have the biggest prevalence among the other trees whereas in periods 2 and 4, the primary cases of the dominant tree do not have the biggest prevalence among others. This indicates the seasonality of the prevalence effects on ARI’s spread in the region. Table 3 provides the basic features of epidemic forest for all periods. We observed that there is no district being the consistent primary case at each period, specifically the dominant tree. Along four periods, each dominant tree has a different root. Period 3 has the most resulting trees and the most child cases at the dominant tree.

**Table 3:** Basic features of the resulting epidemic forest.

Period	Number of trees	Primary case of the dominant tree	Number of child cases at dominant tree
1	8	Palmerah (PAL)	8
2	8	Senen (SEN)	8
3	9	Grogol Petamburan (GP)	14
4	6	Gambir (GAM)	11

**Figure 7:**
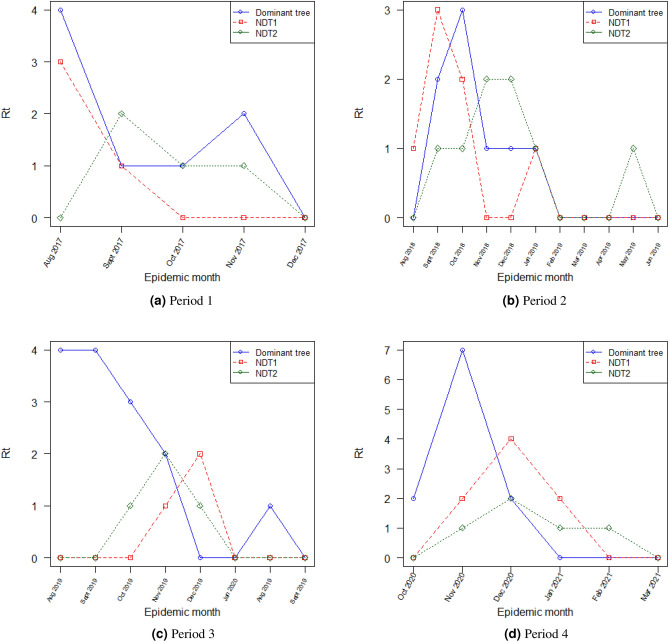
Tree-wise $R_t$s for dominant tree and two selected non-dominant tree for each period.

From the resulting epidemic forest, we calculated $R_t$ for each period at each epidemic month with three spatial scale. Each period has a different onset time which depends on the selected tree. The calculation of $R_t$ started from each onset time. Figure 7 shows three-wise $R_t$ for the dominant tree and two selected non-dominant trees (NDT1 and NDT2) on each period. From Fig. 7 we observe that in general, $R_t$ of the dominant tree in period 1 has a similar trend with period 3 and so as periods 2 and 4 have similar trends. It indicates that the dominant tree can illustrate the character of disease spread of each period and this supports the findings on seasonality prevalence effects on ARI’s spread.

## Discussion

In Indonesia, ARI is considered a health burden particularly for children as its mortality and morbidity rate is relatively high^34^. Bacteria, viruses, and fungi have been considered as etiological agents for ARI^35^. The infections due to bacteria and viruses are transmittable through contact, airborne, droplet, vectors, and vehicular^36,37^. Many variables can affect the transmission of these routes such as environmental factors and crowds of people^36^. The spread of pathogen efficiently happens among humans, particularly through airborne transmission, and may cause an outbreak that has to be controlled^36^.

Studies of ARI’s incidence been conducted mainly focus on surveillance on children in developing countries due to its emergency and limited resource of health support^34,35,38^. The association between several factors such as meteorological factors^39–43^, gender^42^, housing^34,41,42,44^, reliability of healthcare facilities^45^, and chronic diseases^42^, with incidence of ARI which is caused by virus also observed. The incidence was also studied in spatio-temporal aspects to help understand the association of incidence spatially^46–50^. These studies can map the incidence spread in spatial aspects however, they are unable to demonstrate the spread of disease from one region to another. A better understanding of the disease spread between regions may help mitigate and control the outbreak. Few previous studies on ARI’s transmission between regions have been conducted. These studies mainly focus on the spread by human mobility through existing network transportation, that is city network^14^, airline network^27^, and both^51^.

Our research studies the spread of ARI by utilizing the concept of epidemic forest^32^ at a region-scale level. The previous study of epidemic forest which was modeling the disease spread at individual level^32^ can determine the primary cases and the size of spread with different parameter settings. The urgency of determining primary cases at the regional level arises to provide early warning information about the disease’s spread. The objective is to give awareness to health institutions, particularly the health institutions responsible for treating patients in the smallest region-scale, as an attempt to prevent an outbreak.

Aside from being a health burden, an outbreak of ARI can cause social and economic burden^2^. The government should prepare for the costs that arise from it, such as costs for medicine and vaccines. Early warning information given from the resulting epidemic forest can be beneficial for cost management by the government.

The epidemic forest presents a tree-graph model. A tree-graph model is simpler and easier to analyze than a network model. For instance, one possible multiple-to-multiple network model closest to our work presented by^52^. In^52^, more prior knowledge on the construction of the network itself is required, which most are not available in our case, i.e. human mobility. Whilst multiple-to-multiple network models seems to be more realistic, it requires more information in hand compared to epidemic forest model.

The epidemic forest at each period in Fig. 6 provides information on the consistent presence of disease in a certain area. We observe that almost at all periods, the outbreak began at West Jakarta which was marked by early onset time in the area. Based on this result, a suggestion can be made regarding outbreak prevention with priority in West Jakarta.

**Figure 8:**
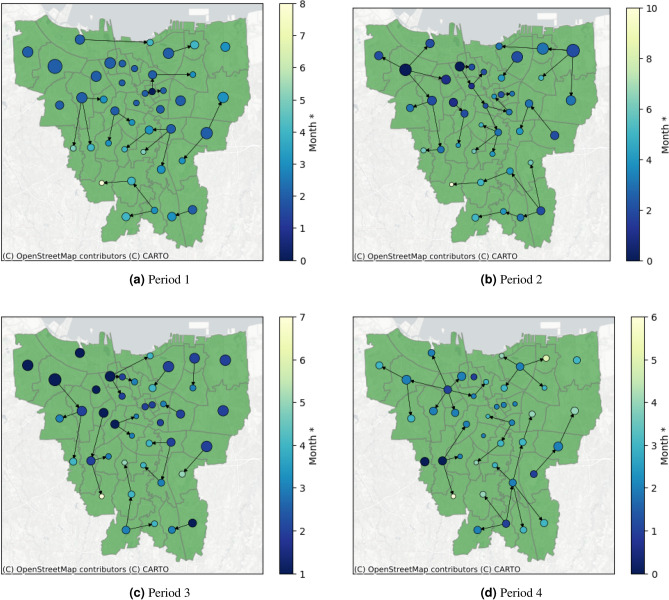
Result of the epidemic forest for all period from simulations.

For illustrative purposes, we conducted a simulation in which we generated an epidemic forest with the condition that the number of cases at the primary case on the dominant tree at the respective period is reduced by 50%. This simulation can provide brief results on whether the prevention of primary cases, which is then represented by a reduction in case numbers, can control the outbreak or not. Table 4 shows the simulation result. We observe that in general, we have more trees compared to the original forest but the number of child cases of the dominant tree is reduced. A significant reduction in child cases of the dominant tree is shown in period 3. By the simulation settings, Tambora district becomes the primary case of the dominant tree in two periods. The resulting epidemic forest from the simulation at each period in Fig. 8 shows that the changing of epidemic forest occurs mainly within the area where the number of cases is reduced whereas another area shows the same trees as the original.

**Table 4:** Basic features of resulting the epidemic forest from simulations.

Period	Number of trees	Primary case of the dominant tree	Number of child cases at dominant tree
1	9	Johar Baru (JB) and Jatinegara (JAT)	4
2	8	Tambora (TAM)	10
3	11	Tambora (TAM)	4
4	6	Grogol Petamburan (GP)	9

Our study has several limitations. We summarize these limitations and future study plans as follows. First, in this study, it is assumed that a region can only be infected by a neighboring region. In reality, there is a possibility that a not-neighboring region can infect a region. We will develop a model that accommodates this possibility in our future research. Second, we set the same threshold value as a marker for the occurrence of an outbreak in each region, without considering the population density of it. Dynamic threshold values for each region are very important in determining a more accurate onset time in respective regions. In future research, we will develop a model with dynamic threshold values for each region. Third, in this study, we did not consider inter-regional connectivity and networks. In the mechanism of the spread of infectious diseases, these two things play an important role because human mobility is a means of transmission^14,27,51^. Aspects of inter-regional connectivity and networks will be one of the model developments in future research. Fourth, our model still uses simple geographic data, i.e., geographic coordinate data to build a network between regions.

Furthermore, we would like to highlight some important points in this paper concerning the tree-like model and the measurement method for determining the distance. In this study, we build the model with the assumption of a single-source infection. We model the spread of the disease between regions as a tree-like model, in which the source of infection is only from one region. To validate this assumption, one interesting future agenda is to collect data on the spread of a disease and do the explanatory data analysis to test our hypothesis. Findings from this approach should either strengthen our results or refine our model to capture the real-world phenomena of the disease spread spatially. On the other hand, identifying this primary source will be very useful for developing countries that may have limited budgets for disease control and management. For these countries, handling outbreaks in the primary source can be a priority so that necessary measures can be applied in the precise area to control the disease spread effectively. In this research, simple spatiotemporal information of the case in the regions is applied to the algorithm. We utilize the centroid-based method to measure the distance between regions for the spatial aspect. However, this method provides an adaptable framework that can incorporate additional data and techniques. For instance, information about human mobility between regions can be used to confirm and enhance the accuracy of the epidemic forest.

## Methods

### The study site

**Figure 9:**
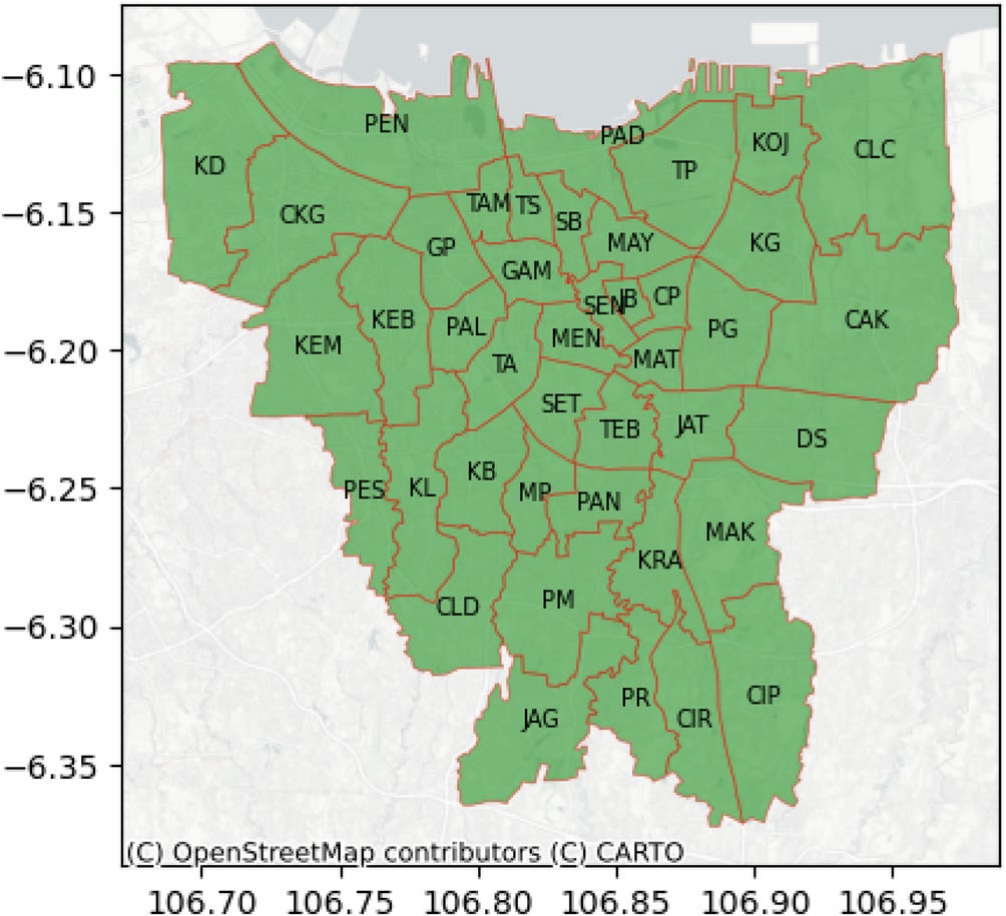
The District of Special Capital Region of Jakarta.

The Special Capital Region of Jakarta is located at $5^{^\circ } 12^{\prime }$S to $6^\circ 22^{\prime }$S and $106^\circ 23^{\prime }$E to $106^\circ 58^{\prime }$E and covers total area of 664.01 square kilometers. The population density in this region is the highest in Indonesia reaching 15,978 people per square meter in 2021. There are 44 districts, 5 cities, and 1 regency under its jurisdiction. Based on the geographic location of the districts, the study site is shown in Fig. 9 using geopandas (v.0.13.2).

### Data collection

Monthly number of pneumonia and influenza infection cases at 42 districts and the number of population in the Special Capital Region of Jakarta are collected from 2017-2021. The data are divided into two sources of health facilities, i.e. hospitals and the district’s center of public health. Three other data which is used as inputs are the geographical coordinates, district adjacency, and district-level population. Geographic coordinates represent the central coordinates of each district, while district adjacency is represented in a symmetric matrix where the diagonal elements are 0 and the off-diagonal elements are either 0 or 1. A value of 1 in the adjacency matrix indicates that the corresponding districts in the row and column are adjacent, while 0 indicates they are not. The geographical coordinates used to build a network between regions. It should be sufficient given that Jakarta is roughly homogeneous with uniformly distributed placement of public transportation. The district adjacency is used to accommodate the assumption that a region is infected by only one neighboring region. Population data is obtained by processing the population density data at the sub-district level.

The population density data at the sub-district level is processed first to obtain district-level population data. For simplification purposes, the population count is assumed to be constant by taking the average value from the population count between 2017 and 2020 for each district. The data for the year 2021 is not included due to data limitations.

### Infectious period identification

The period of occurrence of spikes in cases will be determined for each district. This is accomplished by examining the ratio of ARI cases to the population size. Hierarchical clustering will be applied using the average linkage method and the Euclidean distance metric. This approach aims to group the districts based on their similarities. The results will be presented in the form of a heat map. Visually, the periods with spikes in cases for each district will be identified. Among the three disease data sets, only the Pneumonia data from hospitals shows periods of spikes in cases for each district. Therefore, this data set will be used for the subsequent epidemic forest algorithm.

### Onset of the outbreak

In epidemiology, the beginning of the infectious period of an infected individual is known as onset time^32^. In this research, for the region-scale level, the onset time is determined as the starting point of an outbreak in the particular region that can be identified as the beginning of the peak of cumulative cases with the assumption that there is exactly one single wave of the outbreak for all regions. The data of cases were fitted to the Richards model. The Richards model is shown as the following equation^53,54^:

1 $$\begin{aligned} C(t)=K[1+e^{-r\mu (t-t_i)}]^{-1/\mu } \end{aligned}$$

*C*(*t*) is cumulative number of cases at *t*. Parameters *K* and *r* are the total numbers of cases and the cumulative case number’s growth rate per capita, respectively. The cumulative curve deviation’s exponent is denoted by $\mu$ and the point of inflection on the $x-$ axis is denoted by $t_i$ which shows the timing of changes in downturn or upturn in the growth rate of the cumulative cases number occurs. In this research, the onset of the outbreak for every city is determined by fitting the curve of cumulative cases to the Richards curve to obtain the value of $t_i$.

### Construction of region-scale epidemic forest

An epidemic tree is constructed through an algorithm that generated the possible source of infection of each infected case^31^. The tree consists of nodes and links. The cases are represented by nodes whereas each link represents the parent-child relationship in the infectious process, which is the parent infected the child. The candidates’ parent of a child case are all cases that occur within a certain distance from a child case and are infected prior to the child case. Each child case is assumed to have only one parent. The root of the epidemic tree is called the primary case, which is the first case that arises at the beginning of the spread of disease. The other nodes of the tree are the descendants of the primary case and are called secondary cases. The epidemic forest is built when there are several primary cases^32^.

A child case may have several candidates for its parent. A strength of linkage between these cases is evaluated to eliminate the less-possible candidates. The strength of linkage is calculated based on the spatio-temporal distance between the child case and each of the candidates. In this study, the prevalence of each region is also involved in the calculation. Spatio-temporal distance and prevalence are considered as measures of the possibility of transmission between regions. The transmission may likely to occur when the spatio-temporal distance is smaller and the prevalence between regions is higher. As the three measurements for strength of linkage have different units, we use the scaling method to make them dimensionless so the integration can be done. The equation of the spatio-temporal distance between child case and *j*-th candidate parent are given as follows:

2 $$\begin{aligned} \tilde{D}_{s_j}&=\frac{D_{s_j}}{D_{s_{min}}}\end{aligned}$$

3 $$\begin{aligned} \tilde{D}_{t_j}&=\frac{D_{t_j}}{D_{t_{min}}} \end{aligned}$$

$D_{s_j}$ and $D_{t_j}$ are spatial distance and time difference between child case and *j*-th candidate respectively, whereas $D_{s_{min}}$ and $D_{t_{min}}$ denotes the minimum value of spatial distance and time difference between child case and all candidates of parent case respectively. The spatial distance represents the distance between centroids of each region. The utilization of centroids in determining distances between regions was previously carried out by^55^ to determine the distance between individuals in different cities and^56^ which constructed epidemic trees. As for prevalence is calculated for *j*-th candidate parent by:

4 $$\begin{aligned} \tilde{P_j}=\frac{P_j-P_c}{arg \max \limits _{j}\{|P_j-P_c|\}} \end{aligned}$$

$P_j$ and $P_c$ is prevalence of *j*-th candidate parent and prevalence of child case respectively.

In this research, the concept of the epidemic forest is used to understand the spread of infectious disease at a region-scale. We introduce the algorithm of the construction of region-scale epidemic forest in Algorithm 1.

**Algorithm 1** The algorithm flow of region-scale epidemic forest

**Input**: Onset time, geographical coordinates

**Output**: Epidemic forest

**Steps**:

1. Choose arbitrary case and consider it as child case.

(2) Given the network, determine all neighbouring cases to the child case, namely Candidate 1.

(3) Separate all cases in Candidate 1 which comply with the rule of parent’s onset time, i.e. the onset time of parent must be prior to the child. These cases are called Candidate 2.

(4) Evaluate Strength of Linkage (*SoL*) value of each case *j* in Candidate 2 relative to the child case. The formula of *SoL* is given as follows:
  5 $$\begin{aligned} SoL(\tilde{D}_{s_j}, \tilde{D}_{t_j}, \tilde{P_j})=W_s \cdot \frac{1}{\tilde{D}_{s_j}} + W_t\cdot \frac{1}{\tilde{D}_{t_j}} +(1-W_s-W_t)\tilde{P_j} \end{aligned}$$
  $\tilde{D}_{s_j}$, $\tilde{D}_{t_j}$ and $\tilde{{P_j}}$ as shown in equation (2–4), $\tilde{W}_s$ and $\tilde{W}_t$ are the weight assigned to spatial and temporal component respectively.

(5) Choose a case with the greatest value of *SoL* as a parent for the child.

(6) Repeat steps (1) to (5) for all child cases.

(7) A tree is formed when all child cases have found their parents.

(8) Determine the primary case, i.e., the case that is the initial source of the spread of the disease. In a formed tree, the primary case is the root of the tree.
(9) An epidemic tree is formed when we have more than one primary case.

## Data Availability

The data that support the findings of this study are available from Jakarta Health Office, but restrictions apply to the availability of these data, which were used under license for the current research, and so are not publicly available. Data are, however, available from the authors upon reasonable request and with permission of the Jakarta Health Office.
